# Enhanced transport of nutrients powered by microscale flows of the self-spinning dinoflagellate *Symbiodinium* sp.

**DOI:** 10.1242/jeb.197947

**Published:** 2019-04-24

**Authors:** Zheng Zhu, Quan-Xing Liu

**Affiliations:** 1 State Key Laboratory of Estuarine and Coastal Research, School of Ecological and Environmental Sciences, East China Normal University, Shanghai 200241, China; 2 Shanghai Key Laboratory for Urban Ecological Processes and Eco-Restoration & Center for Global Change and Ecological Forecasting, School of Ecological and Environmental Sciences, East China Normal University, Shanghai 200241, China

**Keywords:** Self-spinning motion, Active fluid, Non-Brownian diffusion, Entrainment effect

## Abstract

The metabolism of a living organism (e.g. bacteria, algae, zooplankton) requires a continuous uptake of nutrients from the surrounding environment. However, within local spatial scales, nutrients are quickly used up under dense concentrations of organisms. Here, we report that self-spinning dinoflagellates *Symbiodinium* sp. (clade E) generate a microscale flow that mitigates competition and enhances the uptake of nutrients from the surrounding environment. Our experimental and theoretical results reveal that this incessant active behavior enhances transport by approximately 80-fold when compared with Brownian motion in living fluids. We found that the tracer ensemble probability density function for displacement is time-dependent, but consists of a Gaussian core and robust exponential tails (so-called non-Gaussian diffusion). This can be explained by interactions of far-field Brownian motions and a near-field entrainment effect along with microscale flows. The contribution of exponential tails sharply increases with algal density, and saturates at a critical density, implying a trade-off between aggregated benefit and negative competition for the spatially self-organized cells. Our work thus shows that active motion and migration of aquatic algae play key roles in diffusive transport and should be included in theoretical and numerical models of physical and biogeochemical ecosystems.

## Abstract (precis)

**Summary:** Many studies suggest that complex and crowded environments slow transport from normal diffusive behavior to subdiffusive behavior, but our findings reveal that diffusivity is still enhanced with a power-law behavior in active microscale flows.

## INTRODUCTION

Motile microorganisms display fascinating spatial patterns and collective behaviors, evoking the focus of attention of biologists, biophysicists and ecologists over the past three decades (Bartumeus et al., 2016; Katija, 2012; Katija et al., 2015; Woodson and McManus, 2007). In particular, the development of tracking techniques has motivated studies on organisms' foraging behavior from macroscale to microscale and nanoscale. Although numerous experimental observations and theoretical models have reported that the active motions of microorganisms have significant effects on their search strategies for resources such as bacteria and algal cells within aquatic environments, most of the studies are still based on a run-and-tumble-like motion behavior of the organisms' themselves (Ariel et al., 2015; Bennett and Golestanian, 2013; Palyulin et al., 2014). As an extension, the entrainment effect of swimming microorganisms leading to enhanced diffusion has recently been introduced in the context of active fluids, including suspensions of swimming bacteria, mixtures of eukaryotic flagellates and ciliary beating (Guasto et al., 2012; Jeanneret et al., 2016; Leptos et al., 2009; Morozov and Marenduzzo, 2014; Shapiro et al., 2014).

Recent experimental results on swarming *Escherichia coli* bacteria revealed an enhanced diffusion of particles through the active motion of cells, namely a thermal bath, as an analog to Brownian motion resulting from molecular collisions (Miño et al., 2011; Wu and Libchaber, 2000). Based on green algae *Chlamydomonas reinhardtii* systems, Leptos and collaborators demonstrated that, in dilute suspensions of the algal cells, the probability density functions (PDFs) of tracer displacements deviate from paradigmatic Gaussianity, manifesting strong exponential tails (Leptos et al., 2009). Unsurprisingly, one may hypothesize that the enhanced diffusion should relate to anomalous non-Gaussian behavior, but the underpinning mechanisms remain largely elusive from experimental points of view (Bechinger et al., 2016; Höfling and Franosch, 2013). Anomalous statistical features have been found in a variety of other biological systems (Caspi et al., 2000; Freedman et al., 2017; Höfling and Franosch, 2013; Tabei et al., 2013; Wang et al., 2009). Limited experimental measurements infer that this non-Gaussian behavior is caused by the flow fields of active swimmers (Drescher et al., 2010; Guasto et al., 2010). However, the physical mechanisms underpinning non-Brownian behavior is lacking at microscale levels. A better biological model still remains to be developed to unravel the underlying mechanisms.

Algal species have evolved ingenious ways to take up nutrients from their aquatic environments. The common bacteria and eukaryotic flagellates use drag-enhancing flagella that help to maintain run-and-tumble behaviors (Jeanneret et al., 2016; Stocker, 2011). A crucial problem for algae is how they take up more nutrients from their local environments in order to compete with the other algal species present. Here, the complex spatiotemporal behavior of individual cells is the result of energy produced by the organisms' rotating flagella (active motility) at the microscopic scale and subsequent cascading of energy toward larger scales that greatly exceed the size of individual organisms for enhanced diffusivity and transport of nutrient particles (Barry et al., 2015; Dabiri and Gharib, 2005; Durham et al., 2013; Kokot et al., 2017; Kurtuldu et al., 2011; Woodson and McManus, 2007). Unraveling the mechanism of enhanced transport may provide deeper insights into the biological processes and sustaining mechanisms of algal blooms (Guasto et al., 2012).

Here, we investigated the statistical behavior of microscale transports on tracer particles in living fluid. Unlike previously reported biological systems such as *E. coli* and *C. reinhardtii* – swimming microorganisms that have remarkable superdiffusive behavior of translational displacement (Ariel et al., 2015; Jeanneret et al., 2016) – we studied a system with self-spinning microalgae, *Symbiodinium* sp. (clade E), that do not exhibit translational displacement and found that the tracer transport still displays a non-Gaussian Brownian motion. The overall displacement behavior is well described by a Gaussian core and exponential tails, owing to the interactions of two mechanisms – the far-field Brownian motion and the near-field entrainment effect along with fluid vortices, which cause the exponential tails’ behavior. The enhanced diffusivity comes from the microscale vortices of spinning cells caused by cell–cell interactions, and the contribution of microscale vortices grows much faster with increased cell concentrations but becomes saturated at the critical cell density of approximately 400 cells mm^−2^ (volume fraction φ ∼4.5%). We also explored this enhanced transport behavior with a simple numerical simulation, and show that the dynamic behavior is well captured by a simple microscale vorticity–diffusion process. Finally, the statistical properties of the transport displacements were studied for tracers in proximity to and far away from the nearest swimmer to give a firmer insight into the enhanced transport patterns in algal species.

## MATERIALS AND METHODS

### Algal cell culture and image acquisition

Free-living *Symbiodinium* sp. (clade E) were cultured in artificial seawater with F/2 medium (Guillard and Ryther, 1962) in a 100 ml flask, and placed in an incubator (INFORS HT Multitron pro, Switzerland). The daily light cycle was 12 h of cool light with an intensity of 2000 lx and 12 h in the dark at 20°C. The algal cells in the experiments were in an exponential phase after 14 days culture and observed on the gas–liquid interface inside a disk-shaped chamber made of silica gel with a plastic gasket. Higher concentrations can be achieved by natural settling approximately 10 times (10–20 min each time) in a long chamber. To study the active diffusion of passive tracers influenced by the active spinning cells, milk colloid (diameter 1∼2 μm, Deluxe Milk, MENGNIU) was added as a tracer to the suspensions (volume ratio 1:200). Visualization was performed under an inverted microscope (Nikon Ti-U) with a 20× magnification objective, and an sCMOS camera (pco.edge 5.5, Germany) was used to capture the motion of cells and the tracers at 50 frames s^−1^ for 90 s. The observation field was approximately 316×316 μm^2^. Image processing was performed using MATLAB 2017a. The trajectories of cells and tracers were extracted from the movies through tracking programs (Zhang et al., 2010).

### Flow-field characterization of the particle image velocimetry

The flow fields of the swimmers were qualitatively characterized by tracking the milk-colloid tracers (because plastic beads will stick together in the seawater). We used PIVlab (Thielicke and Stamhuis, 2014) (version 1.5) to analyze a series of continuous pictures taken at 50 frames s^−1^. Before loading into PIVlab, we used a top-hat transform and masked all of the swimmers as the pre-processing of the images. After obtaining a series of instant velocity fields, we took the average of each rotational period to obtain a mean flow field generated by the spinning of the cells and normalized vorticity by the maximum to denote the orientation of the flow. A positive value indicates counter-clockwise flow whereas a negative value indicates clockwise flow. To characterize the difference between flow states, we used spatial velocity–velocity correlation functions:(1)
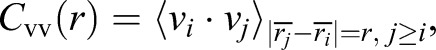
where *v_i_* and *v_j_* are the velocities of the flow, 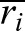 and 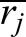 are the positions of the velocity, *i* and *j* are the indices running over all vectors. The normalized *C*_vv_(*r*) curves show a strong negative correlation in the microvortex but decline to zero fast in turbulent flows.

### The near-field and far-field calculations

In order to separate the contribution of the active part from the passive Brownian motion, we set a threshold at ∼35 μm, relating to the spinning center. The length is a bit larger than the sum of the rotating radius and one body size, which conforms to the scale of a micro-vortex. We then recalculated the PDFs of displacement according to the nearest distance of particles and the spinning center, so that the PDF is decomposed into two parts, the near-field part (distance <35 μm) and the far-field part (distance >35 μm).

## RESULTS

### Density-dependent turbulence and active transport

**Fig. 1.**
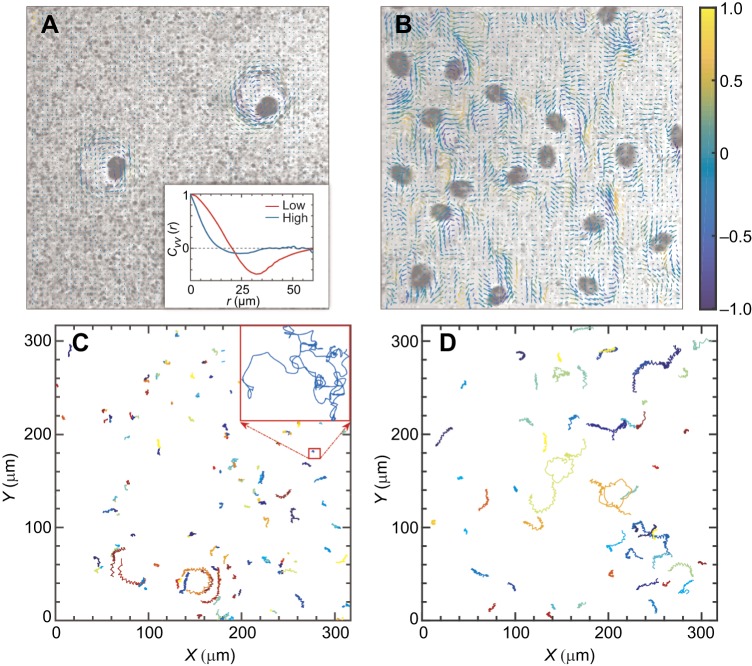
**Experimental observations of the spatial microscale turbulence.** (A) Snapshot of the coarse-grained velocity field generated in one rotation period (≈0.24 s), calculated by PIVlab (see Materials and Methods for details, coarse graining length Δ*l*=2.34 μm), obtained from *in* *situ* measurement with tracers at low algal density (see Movie 1). Inset: spatial velocity correlation functions with low- and high- density cells, respectively. (B) A microscale turbulent state dominates the transport patterns of tracers at high algal densities (same method as used in A). Colored arrows in A and B show fluid flow direction distinguished by normalized local vorticity, 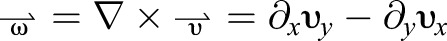. The spatial scale is approximately 165 μm. Color bar indicates the values of the normalized vorticity in A and B. (C,D) Spatial dispersals of tracers at low and high cell density, respectively, where Brownian motion of particles dominates the transport patterns (C), compared with the entrainment effect along with fluid turbulence becoming dominant, which is caused by the active swimmers (D). All trajectories were randomly chosen at 40 s intervals and only a few tracers are shown for clarity.

*Symbiodinium* sp. (clade E) are unicellular with a body size of *d*≈12±3 μm in diameter and possess two dissimilar flagella arising from the ventral side (one longitudinal flagellum and one transverse flagellum) that are responsible for a rotational speed of ω≈25±5 rad s^−1^, according to our laboratory experimental measurements. The transport of nutrients in living fluids without external pressure is composed of intrinsic diffusion and active transport processes induced by microorganisms. The trajectories of particles influenced by microorganisms can be divided into three types: intrinsic Brownian motion, circle-like behavior induced by hydrodynamics and entrainment effects caused by collision with active swimmers. The self-spinning *Symbiodinium* cells can create large vortex patterns if their concentration is sufficiently high (Fig. 1A,B). With increasing algal density, the near-field effect of the vortical flow gradually governs the entire hydrodynamics (Fig. 1). Then, large vortices merge together to form microscale turbulences, where intrinsic Brownian motion effects become less important to nutrient transport compared with the collision-dominated high-density regime (Fig. 1C,D). These microscale turbulences lead to a long-range transport that has profound effects on nutrient mixing and molecular transport in microbiological systems.

Video microscopy reveals that the density of self-spinning cells controls the spatial scale of the vortical flow (inset of Fig. 1A and Movie 1) and the displacement of tracers (Fig. 1C,D) in *Symbiodinium* systems. The coarse-grained mean velocity field averaged by an algal rotation period (approximately 0.24 s) is shown in Fig. 1A,B under low and high density, respectively (see Movie 1). At low density, the spatial correlation *C*_vv_(*r*) of the velocity vectors as a function of the distance *r* has a minimum at *r*≈34 μm, corresponding to the approximate diameter of a vorticity generated by a spinning cell (inset of Fig. 1A), contrasting with a rapid decline and small vortical scale at high density.

**Fig. 2.**
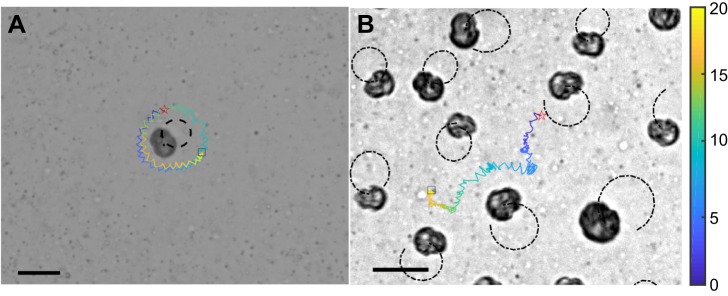
**Density-dependent transport behavior of nutrient particles in microscale flows generated by self-spinning algal cells.** The illustrations show the different behaviors of nutrient particles driven by the near-field hydrodynamics in active living fluids: (A) entrainment motion at low algal density and (B) enhanced transport at high algal density. The dashed circular orbits represent the trajectories of active algal cells with radius *R*, and the colored solid lines describe the trajectories (plotted over 20 s) of nutrient particles in the turbulent flows. The red stars and black squares represent the position of the initial time and the end time, from experimental measurements (see Movie 2). Scale bars: 20 μm and the color bar indicates the time scales.

Based on the velocity analysis of the fluid field, we hypothesized that a single spinning *Symbiodinium* cell can only attract tracers (nutrient particles) around itself (within radius *R*) through a vortical flow, whereas the transport dynamics are enhanced through the collective self-spinning behavior. Experimental results directly support this hypothesis (see Fig. 2 and Movie 2). At high density, the tracers are no longer fixed around one algal cell, but drifted among the algal cells instead. Moreover, in our experiments, a few particles will be pulled towards the algae when the distance is closer than a critical radius *R* (∼30 μm) towards the self-spinning center. It is intuitive that the particle moves much further in the ‘algal bath’ rather than remain fixed around a single cell. This active transport implicates an enhanced diffusion mechanism and uncovers the potential benefits of collective spinning behavior despite the existence of strong intraspecific competition between algal cells.

### Departures from Gaussianity and underlying mechanisms

In order to quantify the displacement behavior of the enhanced transport in living fluids, firstly, we measured the displacement of Δ*x* in the ‘algal bath’ and calculated the PDF of the tracer displacements *P*(Δ*x*,Δ*t*) along an arbitrary direction for a number of increasing time intervals Δ*t*. PDFs of the in-plane tracer displacements coincide with the following formula (Leptos et al., 2009):(2)
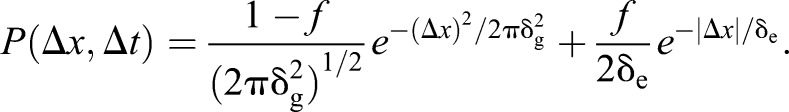
Eqn 2 can be seen as a weighted sum of Gaussian and exponential distributions. *f* describes the contribution of the exponential tails, δ_g_ represents the variance of the Gaussian transport, and δ_e_ represents the characteristic decay length of transport displacements owing to the entrainment effect.

**Fig. 3.**
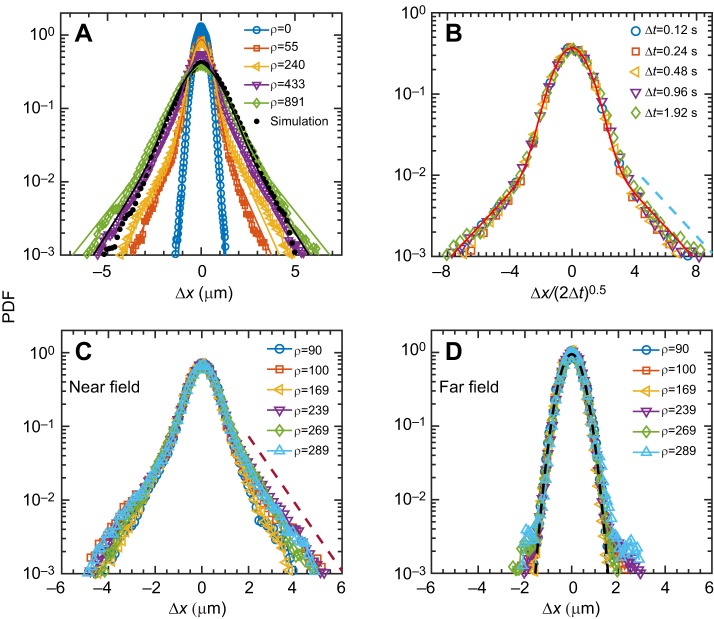
**Probability density functions (PDFs) for tracer displacements.** (A) Ensemble PDFs for various densities (ρ; cells mm^–2^) of active algal cells, showing the broadening of the Gaussian core and emergence of exponential tails. Eqn 1 was used to obtain the relative contribution of the enhanced displacements resulting from the near-field entrainment effect and vortical flows in comparison with far-field Brownian motion. Black dots are the results of the simulation at high density. (B) Diffusive rescaling of PDFs at ρ=90 cells mm^–^^2^ and various time intervals (Δ*t*) shows an exponential-tail behavior with a slope of −0.48, illustrating that data collapse to the master scaling law. (C,D) Overlaid PDFs of displacements of tracer particles associated with various algal densities at a near-field exponential-tail behavior with a slope of −1.05 (C) and at a far-field Gaussian core behavior (D), respectively. All statistical displacements were at a fixed time interval of Δ*t*=0.24 s (a rotational period), except in B.

Without swimmers (*Symbiodinium* cells), tracers (nutrient particles) undergo pure Brownian motion and exhibit accurate Gaussian behavior, whereas with the presence of active spinning cells in the fluid, the exponential tails appear (Fig. 3A). At a fixed interval Δ*t*=0.24 s (the algal rotation period), the PDFs reveal a broadening of the Gaussian core and a growth of the magnitudes of exponential tails with increasing cell density. As shown in Fig. 3B, the PDFs collapse to form a master curve, implying the general scale-law behavior independent of time. This qualitative behavior coincides with previous studies of thin films of swimming bacteria and microalgae displaying a run-and-tumble behavior (Jeanneret et al., 2016; Leptos et al., 2009). However, in contrast to run-and tumble behavior, we show that this exponential tail behavior results from the near-field entrainment effect in our spinning biological systems (Fig. 3C and Fig. S2). With varying densities, the near-field displacement PDFs collapse to a master scaling again, and display the same magnitudes of the exponential tails and the same width of the Gaussian core; the far-field displacement shows the same width as a Gaussian distribution. The results in turn are robust for the various intervals at Δ*t*=1.2 s and Δ*t*=2.4 s (see Fig. S1), although the near-field displacement seems to not approach a Gaussian distribution with increasing time intervals, but it still shows the broadening of the Gaussian core, which indicates that the enhancement of the transport is dominated by the near-field entrainment along with fluid flows driven by the active spinning *Symbiodinium* cells.

### Enhanced transport and diffusive scaling

**Fig. 4.**
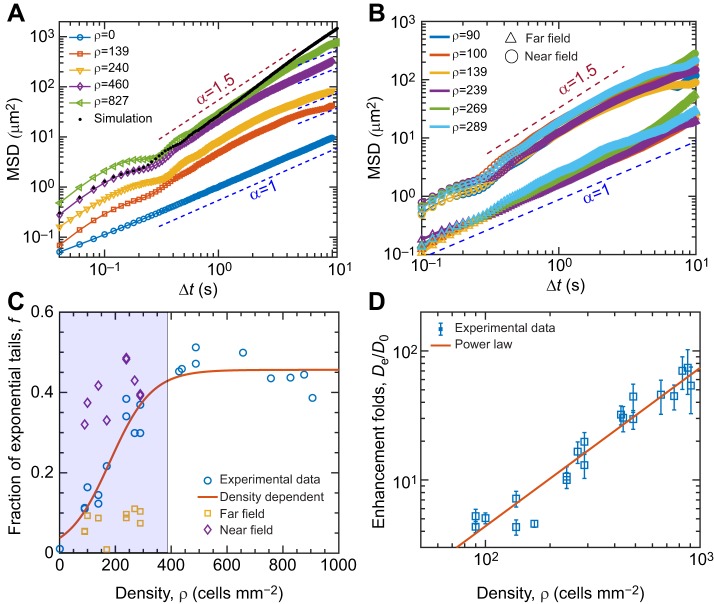
**Enhanced diffusional properties of tracer particles affected by active algal density.** (A) Mean square displacement (MSD) of the tracers versus time at various active algal densities (microscale vortices), showing diffusive scaling behavior with increasing time scales. The motion behaviors shift from Brownian diffusion to super-diffusion with increasing density of algal cells at long-term scales. Black dots are the results of the simulation at high density. (B) MSD of various algae densities separating into near field (circles) and far field (triangles); the statistical distances exhibited a threshold at 35 μm relative to the spinning center. (C,D) Density-dependent enhanced effect of the contribution of exponential tails (C) and the enhancement factor (D) calculated from the PDF behaviors in the two-dimensional experimental data, where the solid curves indicate the nonlinear saturation function and the scaling law of 
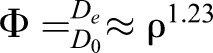
 to fit the experimental data. Error bars in D represent ±s.d. The regression curves in C and D are statistically significant (*P*<0.001) and have *R*^2^ values of 0.933 and 0.938, respectively.

The broadening of the PDFs with cell density indicates significantly enhanced transport. To quantify this behavior, we used the mean square displacement (MSD) of particles for varying cell densities to describe the scaling law of spatial dispersal, with the expression MSD(Δ*t*)=<|*r*(*t*+Δ*t*)–*r*(*t*)|^2^>, where the  brackets denote an ensemble average over thousands of particles. We observed that MSD∼Δ*t*^α^ with α*=*1.0 for the absence of spinning cells at all times, which agrees with expected a normal diffusion behavior. In contrast, if the density increases, the diffusive regime shows an anomalous transport behavior in which the exponent α gradually increases from 1.0 to 1.5 (Fig. 4A).

To elucidate the origin of the observed anomalous diffusion, we further quantified the contribution of the near-field entrainment and far-field motion to MSD and PDFs through the parameters of Eqn 2. As one sees from Fig. 4B, the anomalous diffusion behavior comes from the near-field entrainment effect that is driven by a microscale vortical flow. With the increasing density, the fraction of exponential tails reveals a nonlinear density-dependent relationship (Fig. 4C), and it is saturated at a value of approximately 0.5 for cell densities above approximately 400 cells mm^−2^ (volume fraction φ ∼4.5%). Going beyond this critical density, the nearest distance between the algal cells is close to 35 μm, and the interaction is fully dominated by the entrainment effects along with the near-field hydrodynamics. The continued growth of the spatial decay length implies that the tracers frequently jump from one vortical flow to another with increasing density (Fig. S2). It is obvious that the strength of the entrainment effects also grows with the increasing density of the active cells.

For micro-particles exhibiting Brownian motion in two dimensions, the MSD is proportional to Δ*t*, following MSD∼4*D*_e_Δ*t* for Δ*t*>>τ, where τ is a characteristic correlation time. For pure Brownian motion alone, the diffusion coefficients should be *D*_0_=*k*_B_*T*/6πη*a*, resulting from the Stokes–Einstein relationship of spherical particles through a liquid with low Reynolds number (Einstein, 1905). Here, *k*_B_, *T*, η and *a* are Boltzmann's constant, the absolute temperature, the dynamic viscosity and the particle radius, respectively. Substituting the following values into the equation of *D*_0_, *T*=297.65 K, η=1.2×10^–3^ Pa·s and *a*≈0.75 μm (mean), we can obtain *D*_0_=0.218 μm^2^ s^−1^, which is a good agreement with the experimental value 0.229 μm^2^ s^−1^ underlying the absence of active spinning cells. The effective diffusion coefficients follow a power law with an exponent of 1.23 as active algal density increases (Fig. 4D). This is different in comparison with previous values reported in active bacterial living fluids displaying a linear relationship (Leptos et al., 2009).

**Fig. 5.**
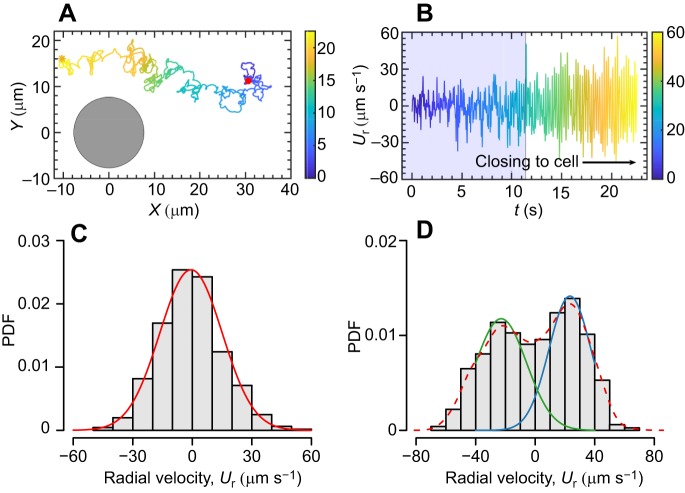
**Dynamic behaviors of tracers within active fluids.** (A) The trajectory of a tracer underlies enhanced diffusion scenarios powered by microscale vortices arising from rotating algal cells. Black disk represents the approximate size of the rotated circle of an algal cell and red dot represents the initiating position of a tracer. (B) The radial velocity of the tracer was dominated by Brownian motion to near-field entrainment transport along with near-field hydrodynamics generated from the rotating cell, where the radial velocity remarkably shifts to a periodic-like oscillation and enlarged amplitude close to the cells. (C,D) Typical unimodal and bimodal distributions at far-field Brownian motion and near-field enhanced diffusion, respectively; the solid curves are Gaussian fits to the radial velocity (*U*_r_) data.

To gain additional insight into entrainment-induced transport in active spinning cells, we explored the movement speed of the tracers along the Stokes flow around a cell. Fig. 5A depicts an experimental trajectory tracking of a tracer, showing the tracer oscillations, powered by the microscale vortices, which are enhanced as the tracer gets closer to the rotating cell in a frame of reference moving with the algal cell. These enhanced amplitudes of velocities suggest a long persistent length and time within a vortex (Fig. 5B) at a high cell density, i.e. trapped by the vortex. Note that the observed trajectories are twisted if the particle is beyond the critical distance, whereas it shows a zig-zag feature of the trajectories at the critical distance (Fig. 2 and Movie 2). The radial velocity of the tracer further confirms that it is dominated by Brownian motion to near-field entrainment transport along with near-field hydrodynamics; and the velocity distributions display typical unimodal and bimodal patterns at far-field and near-field hydrodynamics, respectively.

### Theoretical model

We modeled the passive particles as spheres immersed in the concentration field with active spinning cells. The two-dimensional projection of the stochastic trajectory of a particle in the laboratory experiments, [*X*(*t*),*Y*(*t*)], can be expressed as:(3)
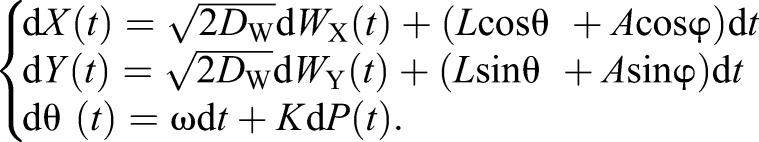


The stochastic equations integrate the standard Wiener processes d*W*_X,Y_(*t*) of Brownian motion with diffusivity *D*_W_ (without active particles), and a small displacement with φ(*t*+*T*)=–φ(*t*), caused by the near-field entrainment effect of the fluid vortices, and finally with a Poisson process for the jumps from one vortical flow of a swimmer to another, d*P*(*t*). *T* denotes a half of the cells' turning period (∼0.12 s). *L* and *A* denote the arc length (per step) caused by the tangential force and the radial force generated by the spinning motion, respectively. These dynamics are simulated using a time step Δ*t*=0.02 s, the same as for the experimental measurements.

Our theoretical simulations suggest that the random walks with diffusivity *D*_W_ have little impact on the final trajectories, because the effective turbulent transport is up to 80-fold larger than the Brownian motion of microparticles in diffusivity. The produced trajectories of model 3 (see Eqn 3) coincide well with the experimental trajectories at high densities (Fig. S3). The simulation predicts that the zig-zag behavior and circular motion around one algal cell will switch to another trapped basin of an algal cell, and is in remarkable agreement with our experimental estimates of *P*(Δ*x*,Δ*t*) and MSD, as shown in Figs 3A and 4A.

## DISCUSSION

Microorganisms play an important role in trophic dynamics and biogeochemistry of marine ecosystems through collective and motile behaviors, as their concentrations and activities often peak at localized hotspots (Doostmohammadi et al., 2012). Generally, negatively buoyant and non-motile cells such as diatoms are dependent on turbulence to keep resuspending them into the photic zone, whereas the motility of dinoflagellates allow the cells to control their vertical position based on resource requirements such as light, which more abundant at the surface and in nutrient-rich areas (Ross and Sharples, 2007). *Symbiodinium* cells are thought to have lower photosynthetic rates and higher metabolic costs and nutrient affinity coefficients than diatoms (Smayda, 1997), and they have evolved a unique motility behaviour for symbiosis with coral reefs. Numerous algae species possess different motility patterns and survival strategies for coping with nutrient-depleted conditions (Jeong et al., 2012). However, besides foraging for nutrients directly, hydrodynamic cell–cell interactions, which grow more relevant as the cell density increases, have been ignored thus far in numerous studies (e.g. Breier et al., 2018; Lauga et al., 2009).

Here, we have shown that the statistics of passive-tracer displacements in suspensions of spinning microalgae *Symbiodinium* sp. (clade E) exhibit a Gaussian core with exponential tails owing to the near-field entrainment effect driven by the vortical flows. This argument is supported by both experimental and theoretical results with extracted tracers close to the active spinning cells. Through calculation of the effective diffusion coefficients, we found that an effective turbulent transport is up to 80-fold larger than the Brownian motion of microparticles in seawater, and the contribution of microscale vortices grows much faster with increased algal density but becomes saturated at the critical density of approximately 400 cells mm^−2^ (volume fraction φ ∼4.5%). The integrated model with the far-field Brownian motion and the near-field entrainment effect further implicates the enhanced transport behavior of particles at a high algal density.

In our experiments, the *Symbiodinium* sp. concentration ranged from 0% to 10%, much higher than previous experiments on the green alga *Chlamydomonas reinhardtii* (Leptos et al., 2009; Jeanneret et al., 2016) with a maximum of approximately 2.2%, which can account for the appearance of the saturation. At the critical density, the mean distance of the nearest neighbor is close to 40 μm, which almost equals the microvortex scale – 34 μm – a *Symbiodinium* sp. cell can generate. Below the critical density, the random walk of the nutrient would dominate the system because the distance is too large for *Symbiodinium* cells to interact with each other. Moreover, the maximum concentration in culture can only increase up to 5–6% – a bit larger than the critical value. This may suggest a trade-off between collective behaviors of enhanced transport and individual uptake within the flows.

Compared with existing studies on microalgae (Jeanneret et al., 2016) and bacterial (Jeanneret et al., 2016) and synthetic microswimmers (Kokot et al., 2017) performing ‘run-and-tumble’ behaviors in suspension, the entrainment effects and enhanced transport mainly arise from the collisions between the microparticles and the swimmers. However, our experimental results showed that the enhanced transport could originate from stable vortical flows generated by spinning behavior. This stable vortex would make a nematic arrangement to embed the largest number of algal cells, but minimize the cost of energy consumption. Moreover, because of the spinning feature of the algal cells, the entrainment effect generates long-range transport more easily at a high density rather than a low density.

## Supplementary Material

Supplementary information
