# Supplementary material for: Enhanced transport of nutrients powered by microscale flows of the self-spinning dinoflagellate Symbiodinium sp
Source: J Exp Biol. 2019 Apr 24;222(8):jeb197947. doi: 10.1242/jeb.197947 (PMC6503948; doi:10.1242/jeb.197947)
Supplement: Supplementary information [file jexbio-222-197947-s1.pdf]

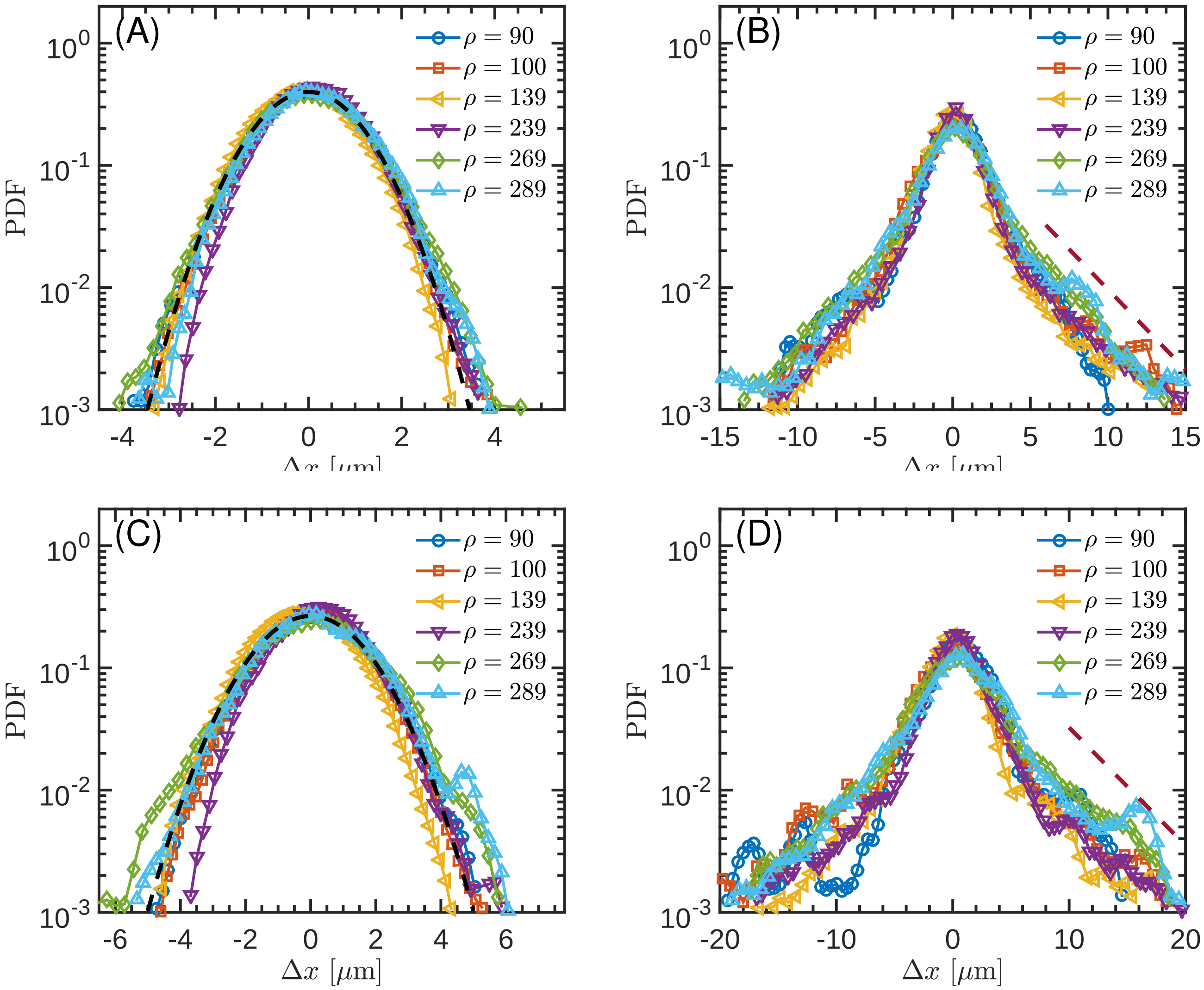


**Fig. S1. Probability density functions (PDFs) for tracer displacements.** Overlaid PDFs of displacements of tracer particles associated with various algal densities on far-field Gaussian core behavior (A, C) and near-field exponential-tails behavior (B, D) with a slope $k = -0.30$ and $k = -0.22$ respectively. The statistical distances take the threshold at 35 $\mu$m (about 2-fold body sizes) relative to the center of spinner circles. (A, B) Time interval $\Delta t = 1.2$ sec and (C, D) $\Delta t = 2.4$ sec.

Journal of Experimental Biology • Supplementary information

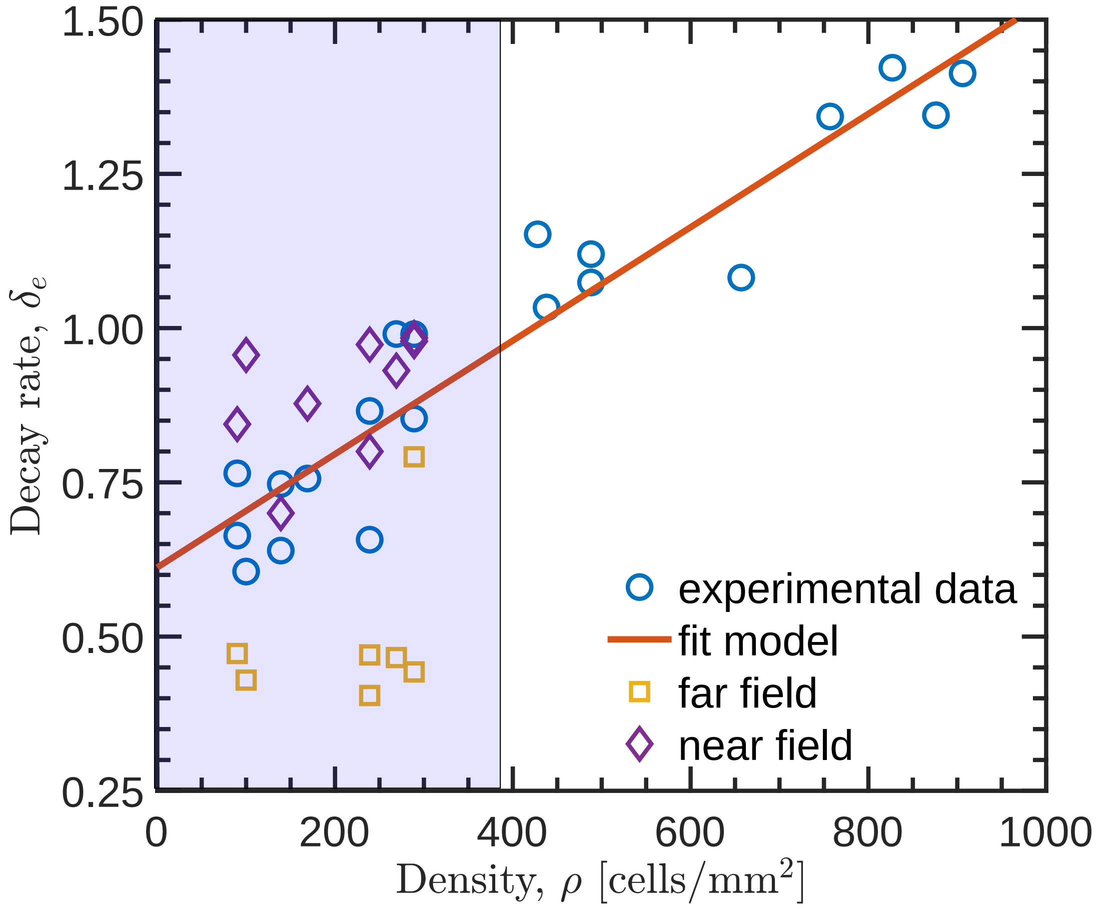


**Fig. S2. Spatial decay rates of turbulence caused by the 'algal bath'.** The relationship of decay rate of long exponential-tail PDFs with increasing packing density of active algal cells. The solid line represents a best linear fit to the data with a slope 7.689. The regressions curve is statistically significant ($p < 0.001$) and has $R^2$ value corresponding to 0.900.

Journal of Experimental Biology • Supplementary information

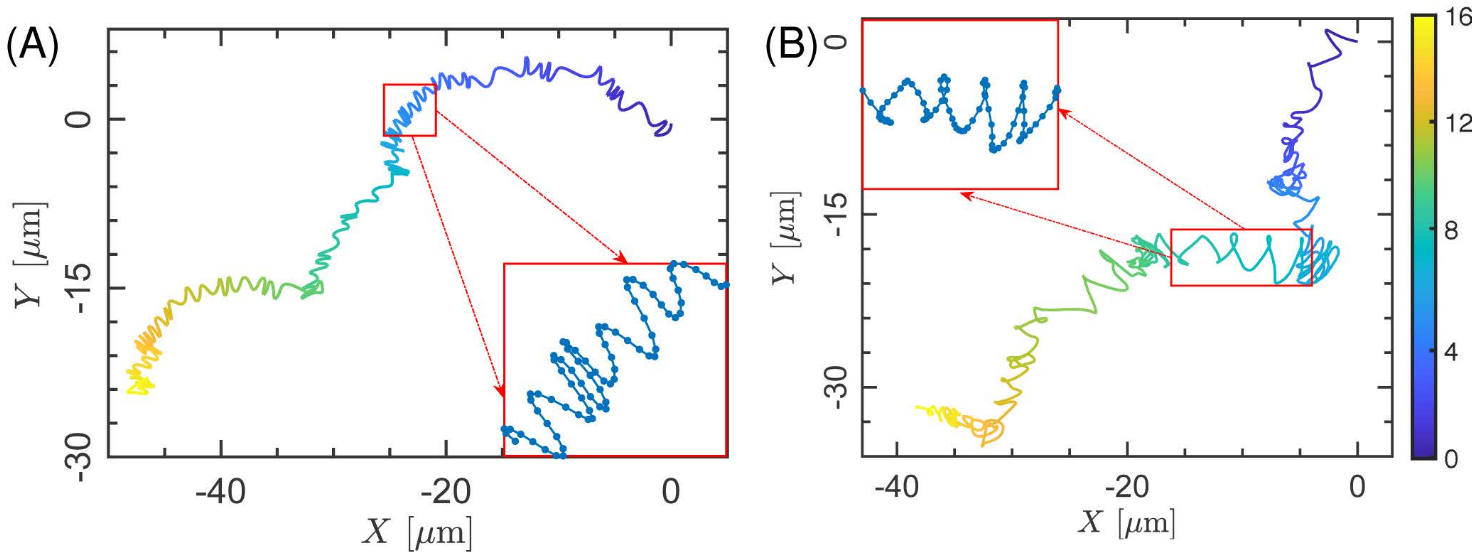


**Fig. S3. Trajectories at high algal density.** (A) The results of simulation solved by the stochastic differential equation (2) comparison with the real experiment trajectories (B). The model well captures the transport behavior, and suggests that the enhanced transport understand with the interage of Brownian motion and Poisson events entrainments. The colorbar describes the time scales of the experimental measurement and theorecal simulation.

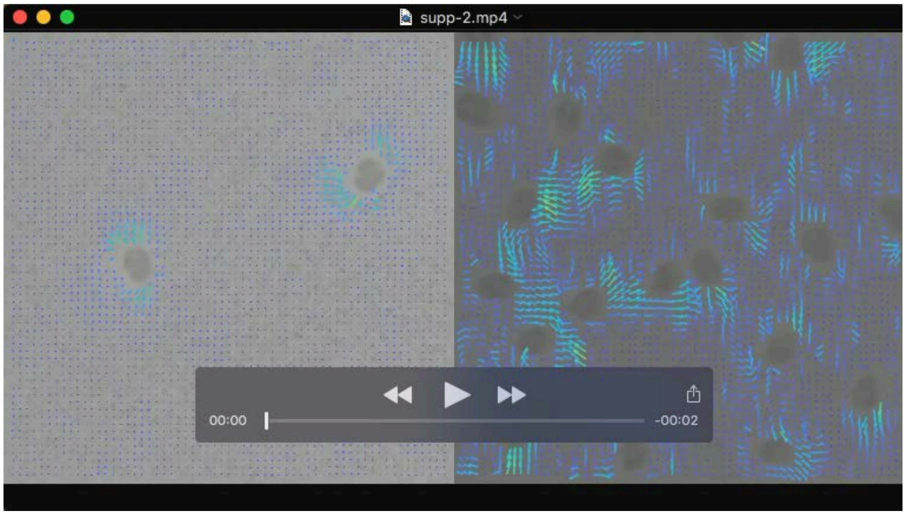


**Movie 1.** Instant velocity field analyzed by PIVLAB package. The size of the visual fields is 165 $\mu$m by 165 $\mu$m and the coarse graining length $\Delta l$=2.64$\mu$m. The video is played 3.5 times slower. (left) and (right) corresponding to Fig. 1(A) and (B) in the main context.

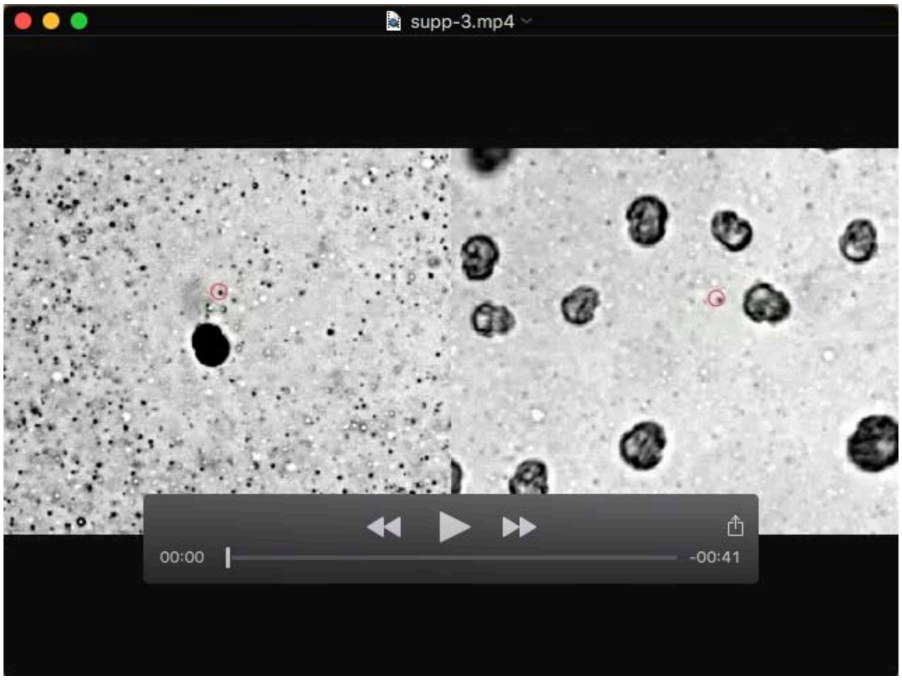


**Movie 2.** Tracer's trajectories influenced by the swimmers. At low algal density (left, two times slower) in contrast to the high algal density (right, normal speed).

Journal of Experimental Biology • Supplementary information
